# Production of the SARS-CoV-2 Spike protein and its Receptor Binding Domain in plant cell suspension cultures

**DOI:** 10.3389/fpls.2022.995429

**Published:** 2022-10-21

**Authors:** Bárbara A. Rebelo, André Folgado, Ana Clara Ferreira, Rita Abranches

**Affiliations:** Plant Cell Biology Laboratory, Instituto de Tecnologia Química e Biológica António Xavier (ITQB NOVA), Universidade Nova de Lisboa, Oeiras, Portugal

**Keywords:** molecular farming, plant cell packs, *Medicago truncatula*, tobacco BY-2 cells, COVID-19, recombinant protein

## Abstract

The COVID-19 pandemic, caused by the worldwide spread of SARS-CoV-2, has prompted the scientific community to rapidly develop efficient and specific diagnostics and therapeutics. A number of avenues have been explored, including the manufacture of COVID-related proteins to be used as reagents for diagnostics or treatment. The production of RBD and Spike proteins was previously achieved in eukaryotic cells, mainly mammalian cell cultures, while the production in microbial systems has been unsuccessful until now. Here we report the effective production of SARS-CoV-2 proteins in two plant model systems. We established transgenic tobacco BY-2 and *Medicago truncatula* A17 cell suspension cultures stably producing the full-length Spike and RBD recombinant proteins. For both proteins, various glycoforms were obtained, with higher yields in Medicago cultures than BY-2. This work highlights that RBD and Spike can be secreted into the culture medium, which will impact subsequent purification and downstream processing costs. Analysis of the culture media indicated the presence of the high molecular weight Spike protein of SARS-CoV-2. Although the production yields still need improvement to compete with mammalian systems, this is the first report showing that plant cell suspension cultures are able to produce the high molecular weight Spike protein. This finding strengthens the potential of plant cell cultures as production platforms for large complex proteins.

## Introduction

The COVID-19 pandemic, caused by the SARS-CoV-2 coronavirus, has been an opportunity for scientists to work together as a community toward developing rapid solutions to fight an emerging problem. Joint research and immediate sharing of results in various areas, from diagnostic tools to therapeutic solutions, have contributed to preventing a virtually unknown number of fatalities worldwide. Since the start of the pandemic, the scientific community focused on the use of standard and fast production platforms, such as bacterial systems, to express functional SARS-CoV-2 proteins. However, it was rapidly realized that these species would be laborious and limited in producing the required complex viral proteins. Thus, animal cells promptly became the expression system of choice. In the meantime, several groups within the plant community joined efforts toward the rapid production of reagents and therapeutics to help combat COVID-19 (He et al., 2021; Lobato Gómez et al., 2021), contributing to the strengthening of eukaryotic platforms for the expression of glycosylated viral proteins.

Almost all reported work on plant-based recombinant products for COVID-19 is on transient expression in *Nicotiana benthamiana*. Reported recombinant proteins include Receptor Binding Domain (RBD), Spike (S) protein or its subunits (S1 and S2), the nucleocapsid (N), membrane (M) and envelope (E) viral proteins, antibodies, and the angiotensin converting enzyme 2 (ACE2) receptor. This information is compiled in **Table 1**.

**Table 1:** Reported Plant-made COVID-19 related proteins (March 2020 to July 2022).

Transient expression (*Nicotiana benthamiana*)		
**Receptor Binding Domain (RBD)**	Ceballo et al., 2022 Diego-Martin et al., 2020 Khorattanakulchai et al., 2022 König-Beihammer et al., 2022 Makatsa et al., 2021 Maharjan et al., 2021 Mamedov et al., 2021 Mardanova et al., 2021 Rattanapisit et al., 2020 Rattanapisit et al., 2021 Royal et al., 2021 Schwestka et al., 2021 Siriwattananon et al., 2021
**Spike protein**	Jung et al., 2022 Margolin et al., 2022 Ward et al., 2021
**S1 domain**	Makatsa et al., 2021
**SARS-CoV-2 M, E and N protein**	Moon et al., 2022 Williams et al., 2021
**ACE2**	Mamedov et al., 2021 Siriwattananon et al., 2021
**Antibodies**	Diego-Martin et al., 2020 Jugler et al., 2022 Kallolimath et al., 2021 Rattanapisit et al., 2020 Shanmugaraj et al., 2020
Stable transformation (Lettuce)		
**ACE2**	Daniell et al., 2022

Previously, during the first SARS-CoV epidemics in 2005, a few reports were also published on plant-based systems. Production of recombinant SARS-CoV nucleocapsid was performed by transient expression in *N. benthamiana* leaves (Zheng et al., 2009); expression of recombinant SARS-CoV Spike protein in plants for the production of oral vaccines was also carried out (Pogrebnyak et al., 2005; Li et al., 2006). However, these studies focused only on a subunit of the Spike protein rather than the full-length sequence. In any case, the relatively short duration of the viral disease led to discontinued work in this area.

To date, one relevant plant-made product has already been authorized for human use, the Covifenz^®^ COVID-19 vaccine approved by Health Canada. This vaccine is manufactured by Medicago Inc. and is composed of recombinant Spike glycoprotein expressed as virus-like particles (VLPs) co-administered with GSK’s pandemic adjuvant. The recombinant protein is produced in *N. benthamiana* by transient expression (medicago.com/en/press-release/covifenz/).

While transient expression offers the advantage of being able to respond to an urgent need, it requires the maintenance of a large number of adult plants and continuous agroinfiltration of plant leaves. An alternative to this platform is the use of plant cell suspension cultures, which can be scaled up and grown in a controlled and contained way, producing the desired molecules in a continuous bioreactor. In fact, the first biopharmaceutical approved for human use, Elelyso^®^, is manufactured in carrot cell cultures (elelyso.com). Plant cell suspension cultures are reliable systems for the production of recombinant proteins following GMP requirements (Santos et al., 2016).

One of the COVID-19-related products that has taken much attention due to its high potential in many applications is the Spike (S) protein. Spike is a large glycoprotein (approximately 140 kDa, 180 kDa with glycans) that forms a homotrimer of around 540 kDa and mediates binding to host cells *via* interactions with the human receptor ACE2. SARS-CoV-2 Spike contains two subdomains (S1 and S2) and a conserved furin recognition site at the S1/S2 junction. This site is not present in SARS-CoV (Coutard et al., 2020) and enables proteolytic processing into S1 and S2 subunits.

At the start of the pandemic, Florian Krammer´s Lab in NY developed an effective serological assay to detect SARS-CoV-2, using both RBD and a soluble form of the S protein with some modifications (Amanat et al., 2020). In this modified version of the S protein, the furin cleavage site was removed (RRAR to A), and two point mutations (K986P and V987P) were added to increase stability (Wrapp et al., 2020). The transmembrane and cytoplasmic domains of the S protein were replaced by a thrombin cleavage site and a T4 foldon sequence for trimerization (Amanat et al., 2020).

In this work, we have modified the sequences of soluble Spike and RBD used in Krammer’s work (Amanat et al., 2020) into plant codon optimized versions, which have been cloned into appropriate vectors for expression in plant cell suspension cultures. We have produced recombinant Spike and RBD proteins, in cultured cells of two model species, *Nicotiana tabacum* BY-2 and *Medicago truncatula* A17, and demonstrated that plant cells can secrete a large protein into the culture medium. This is the first report of a stable system to produce COVID-19-related proteins in a continuous and scalable way. Our goal is to obtain the antigens in the cell medium, which can then be purified and used for research studies and clinical applications.

## Materials and methods

### Plant material and plasmid constructs

*Calli* and cultured cells of *Nicotiana tabacum* cv. Bright Yellow 2 (BY-2) and *Medicago truncatula* cv. Jemalong A17 were maintained as described in previous reports from our group (Santos et al., 2019; Rebelo et al., 2020, for Medicago and BY-2, respectively).

We have used the nucleotide sequences of the stabilized version of the Spike protein reported in Amanat et al. (2020) (GeneBank: MN908947.3) and the RBD coding sequence (aminoacids 319-541) reported in the same work, which were further modified by codon optimization for plant cell expression (see **Supplementary Material S1**). Codon optimization for tobacco was performed by GeneCust (www.genecust.com/en/), as well as gene synthesis with the addition of NcoI and SalI restriction sites at the 5´and 3´ends respectively, for both genes. The genes coding for Spike and RBD were separately cloned into the pTRA vector (kindly offered by Thomas Rademacher, Germany) using the NcoI and SalI sites. This resulted in plasmids pTRA-Spike and pTRA-RBD, respectively. The gene cassettes contain the Cauliflower Mosaic Virus 35SS constitutive promoter, a 35S terminator, the selection marker kanamycin under the control of nopaline synthase (nos) promoter, as well as regulatory sequences for enhanced expression and stabilization, and a 6-histidine tag for purification. The binary plasmids were transferred into *Agrobacterium tumefaciens* strain GV3101 pMP90RK by the freeze-thaw method (An et al., 1989).

### Transient expression in tobacco and Medicago plant cell packs

Transient expression in tobacco BY-2 and Medicago cell cultures was performed as described by Rademacher et al. (2019), with slight modifications. For Medicago cell cultures, some adaptations to the protocol used for preparation and infiltration of BY-2 cell packs were necessary, as Medicago cultures form cell aggregates and thus cell packs tend to retain more air. Medicago cells at day 7 of growth, with a packed cell volume (PCV) of 20% (v/v), were transferred into receiver columns (16341616, Macherey-Nagel™), allowed to settle for 10 min, and then vacuum was applied at a pressure of 50,000 Pa for 60 s. This process was repeated until Medicago uniform cell packs were obtained. For BY-2, 1 mL of 5-day-old cultured cells with a PCV of 30% (v/v) was used. Both cell packs were infiltrated with 500 µL of recombinant Agrobacterium carrying the RBD or Spike gene. Cell packs were kept in the dark for 4 days, at 22-24°C in a humid chamber (a closed box lined with wet paper), and collected for further analysis.

### Stable transformation of BY-2 and Medicago cell cultures

Four-day-old tobacco BY-2 cultures were transformed by co‐cultivation with recombinant *A. tumefaciens* as described by An (1985), with slight modifications as described previously (Folgado and Abranches, 2021). Medicago cultures were transformed following a protocol previously established by our group (Santos et al., 2019). Two days after co-culture, tobacco cells were transferred to MS (Duchefa) solidified medium (Rebelo et al., 2020) and Medicago cells were transferred to CIM solidified medium (Santos et al., 2019). Both media contained 0.4% (w/v) of gelrite (Duchefa) and were supplemented with 500 mg/L ticarcillin disodium/clavulanate potassium (Timentin, Duchefa) to eliminate Agrobacterium and 100 mg/L kanamycin to select positive transformants.

### Detection of recombinant proteins

For both transient and stable expression in tobacco BY-2 and Medicago cell lines, cells were paper-filtered and macerated in liquid nitrogen using a mortar and pestle. The extraction buffer used for sample homogenization was either 40 mM phosphate- or 100 mM Tris-based (the latter containing 10 mM ascorbic acid), pH 8 and 7.5 respectively, and was incubated for 1 h at 4°C. Protein extract was added to the sample buffer and boiled for 10 min.

The spent medium samples for RBD from BY-2 cultures were briefly centrifuged. For Medicago cultures producing the RBD protein, the spent medium was concentrated with ice-cold ethanol. The spent medium of BY-2 producing the recombinant Spike protein was adjusted to pH 8 before centrifugation for 1 h, 4°C at 42,000 rpm (Type 45 Ti Fixed-Angle Titanium Rotor, Ultracentrifuge Optima LE-80K, Beckman Coulter). The supernatant was then concentrated by centrifugation (Allegra X-12R, Beckman), 8°C at 3,000 *g*, with an Amicon Ultra-15 centrifugal filter with a 50 kDa cutoff (Millipore). A similar protocol was followed for Medicago lines producing Spike; the spent medium was collected and concentrated with a Vivaspin^®^ 6 centrifugal filter with a 100 kDa cutoff. Sample buffer was added to all supernatants and samples were boiled for 10 min.

For RBD protein analysis, samples were resolved using a 12.5% SDS-PAGE in a Mini-PROTEAN^®^ 3 Cell (Bio-Rad) and transferred onto a nitrocellulose membrane by semi-dry transfer (Transblot Turbo, Bio-Rad). For the Spike protein, samples were separated by 8% SDS-PAGE and transferred to a PVDF membrane using wet tank transfer. Membrane blocking was performed with 5% (w/v) skimmed milk powder and 3% (w/v) BSA prepared in PBS-T for 1 h at RT with agitation. Blots were incubated in anti-RBD (1:2,000, 40592-T62, Sinobiological), anti-Spike (1:2,000, 40591-MM42, Sinobiological), or anti-His tag (1:2,500, A00186, GenScript) overnight at 4°C with agitation, all prepared in PBS-T. Membranes were washed in PBS-T and the corresponding secondary antibodies were incubated for 2 h at RT with agitation. Secondary antibodies used were the following: HRP conjugated anti-rabbit (1:20,000, AS09 602, Agrisera), StarBright Blue 700 anti-rabbit IgG (1:10,000, 12004162, Bio-Rad), HRP conjugated anti-mouse (1:4,000, A3562, Sigma-Aldrich) and StarBright Blue 700 anti-mouse IgG (1:10,000, 12004159, Bio-Rad). The blots were washed, and the signal was detected by chemiluminescence using ECL™ Bright (AS16 ECL-N-100, Agrisera) in a ChemiDoc™ XRS+ System (Bio-Rad) or by chemiluminescence or fluorescence in iBright™ FL1500 Imaging System (Invitrogen™). The protein molecular weight marker was NZYColour Marker II (NZYTech, Portugal).

For the analysis of the S protein in a Blue Native polyacrylamide gel electrophoresis (BN-PAGE), the BY-2 spent medium was collected and treated as described above. A Bis-Tris protein gel was used (5-15%) and 40 μL of each sample were loaded into the gel following the addition of 0.1% Coomassie G-250 and 0.1% non-ionic detergent DDM to the samples. The gel was run at 4°C, for 150 min at constant 6 mA. The cathode buffer (15 mM Bis-Tris, 50 mM Tricine, pH 7) contained 0.02% (w/v) Coomassie G-250 while the anode buffer contained 50 mM Bis-Tris at pH 7. The molecular size of the proteins was estimated using a Calibration Kit High Molecular Weight for Electrophoresis (17-0445-01, Cytiva). BN-PAGE gel was stained with silver nitrate.

### Purification of recombinant Spike protein from tobacco BY-2 cell lines

Tobacco BY-2 cells producing the Spike protein were paper-filtered on the fifth day of growth. The cells recovered through filtration were frozen and lyophilized for 72 h prior to maceration with a mortar and pestle. Per gram of macerated cells, 20 mL of Extraction Buffer (50 mM Tris-HCl, pH 8, 150 mM NaCl, 40 mM imidazole (Millipore), 1 mM TCEP (Sigma) was added. One tablet of a protease inhibitor cocktail (A32965, Thermo Scientific™) was also added, per each 100 mL of buffer. The extract was homogenized at 4°C, after which the protein supernatant was recovered through centrifugation at 16,000 *g* for 20 min at 4°C. Sodium chloride and imidazole were added until the final concentration of 500 mM of sodium chloride and 40 mM of Imidazole, pH 7.6 was obtained. Afterward, the supernatant was recovered by ultracentrifugation at 42,000 rpm for 1 h at 4°C and filtered through a 0.22 μm PES filter (Sarstedt). All buffers used in the purification process were filtered with a 0.45 μm MCE membrane (Filter-Lab).

Purification of Spike protein was performed and monitored by IMAC using an AKTA system (GE Healthcare, USA). Briefly, 85 mL of protein extract was loaded onto a nickel affinity HisTrap™ HP 5-ml column (GE Healthcare, USA) previously equilibrated with 20 mM of phosphate buffer with 500 mM NaCl, 40 mM imidazole, pH 7.5. Unbound material was removed through washing steps using the same buffer. The bound proteins were eluted in the same buffer with a stepwise increase of imidazole, from 80 mM to 500 mM at a flow rate of 5 ml/min. Positive fractions for Spike were pooled and passed through a PD-10 desalting column (Cytiva) for buffer exchange and the fractions were eluted with 86, 132 and 224 mM of imidazole in 20 mM of phosphate buffer with 150 mM NaCl, pH 7.5. The fractions were concentrated 17-fold with an Amicon Ultra-15 centrifugal filter with a 50 kDa cutoff and analyzed by SDS-PAGE and western blot with anti-Spike antibody, as described previously.

### Glycosylation analysis

Samples for MS analysis were prepared as described in Folgado and Abranches (2021). The glycosylation profile of the Spike protein was assessed using LC-MS analysis of glycopeptides, following the procedure described in Castro et al. (2021). Characterization of the recombinant Spike protein was performed using an X500B-QTOF mass spectrometer (SCIEX) connected to an ExionLC AD UPLC system and Protein Pilot Software v. 5.0 (Sciex). The generated mass spectra were processed and searched against SwissProt (reviewed) and Trembl (unreviewed) protein sequences for Viridiplantae and *Nicotiana tabacum*. Peptide identification was considered above 95% of confidence. Glycopeptides were analyzed by LC-MS using the previously mentioned spectrometer. LC separation was achieved through reversed-phase chromatography using an XBridge BEH C18, 2.5 µm 2.1 × 150 mm (Waters). Separation was performed at 200 μL/min with 0.1% formic acid in water LC-MS grade as solvent A and 0.1% formic acid in acetonitrile as solvent B, and the column temperature was set to 40°C. The LC gradient was as follows: 0–5 min, 1% B; 5–50 min, 1–35% B; 50–55 min, 35–90% B; 55–56 min, 60–90% B; 56–60 min, 90% B; 60–62 min, 90–1% B; 62–64 min, 1% B. Peptides were sprayed into the MS through the twin sprayer ion source with the following parameters: 50 GS1, 50 GS2, 30 CUR, 5.5 keV ISVF, 450°C TEM, 80 V declustering potential and 10 V collision energy. An information dependent acquisition method was set with a TOF-MS survey scan of 350–2,000 m/z for 250 ms. MS data were analyzed using the BioPharmaView software (BPV, Version 3.0, SCIEX) and the protein sequences of Spike (Amanat et al., 2020). For glycan identification, N-glycans described in Watanabe et al. (2020) plus the glycans identified in plants (Strasser et al., 2008; Nagels et al., 2012; Castilho et al., 2014) were added to the BPV software database (provided in **Supplementary Material S2**), resulting in a database with 69 glycan structures. Glycans were identified using MS1 data (considering a peptide deconvolution tolerance and m/z tolerance of 5 ppm) and fragmentation data when available (considering an MSMS tolerance of 0.03 Da). For glycans identified only with MS1, data were manually examined for consistency in retention time information and spectrum quality.

## Results

### RBD and Spike were successfully produced in BY-2 and Medicago cell packs

**Figure 1:**
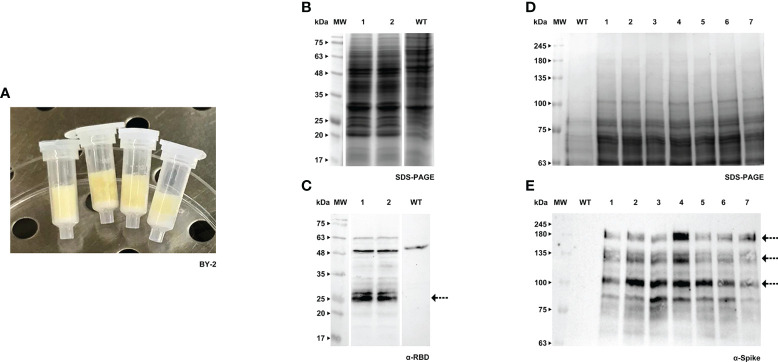
Transient expression of RBD and Spike in BY-2 cell packs. **(A)** Receiver columns with BY-2 cells. **(B)** Stained gel containing total protein extract of two independent BY-2 cell lines infiltrated with pTRA-RBD, WT - wild type. **(C)** Corresponding western blot (WB) with anti-RBD antibody. **(D)** Stained gel containing total protein extract of seven independent BY-2 cell lines infiltrated with pTRA-Spike, WT - wild type. **(E)** Corresponding WB with anti-Spike antibody.

**Figure 2:**
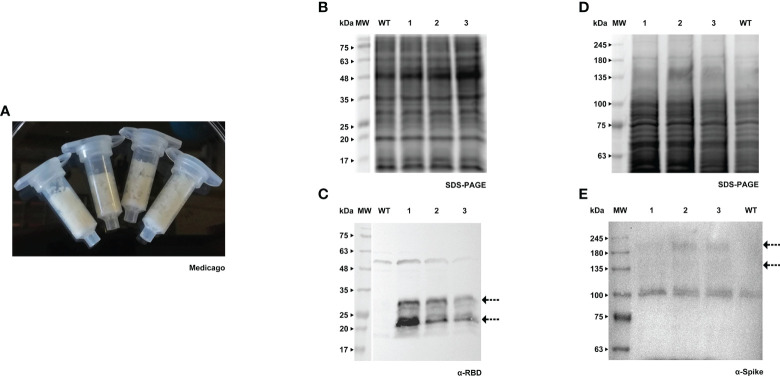
Transient expression of RBD and Spike in Medicago cell packs. **(A)** Receiver columns with Medicago A17 cells. **(B)** Stained gel containing total protein extract of three independent Medicago cell lines infiltrated with pTRA-RBD, WT - wild type. **(C)** Corresponding WB with anti-RBD antibody. **(D)** Stained gel containing total protein extract of three independent Medicago cell lines infiltrated with pTRA-Spike, WT - wild type. **(E)** Corresponding WB with anti-Spike antibody.

RBD and Spike proteins were firstly produced in a transient manner, using the method developed by Rademacher and colleagues (Rademacher et al., 2019). This procedure known as Plant Cell Packs is based on the use of cell suspension cultures from which the liquid medium has been removed. For BY-2 we used the original protocol, but in the case of Medicago it needed adjustments (namely through the modification of time and vacuum pressure applied) since this culture tends to form aggregates in which the air is trapped. The distinct texture of the cell packs of BY-2 and A17 in the receiver columns can be easily observed in **Figures 1A**, **2A**.

Both RBD and Spike proteins were successfully produced in the two species (**Figures 1B–E**, **2B–E**), although a high level of degradation was detected in the case of Spike expressed in tobacco cell packs (**Figure 1E**). This may be caused by the long period of incubation of the cell packs post-infiltration in which endogenous proteases present in BY-2 cultures can cause degradation of the target products. This degradation was not observed in Medicago cell packs. This is in accordance with our previous finding that Medicago cultures present a lower proteolytic activity than BY-2 (Santos et al., 2018).

When comparing the recombinant proteins produced by both species, differences in band patterns can be observed, likely corresponding to distinct glycoforms. For Spike, two similar bands were detected, around 135 kDa and 180 kDa (**Figures 1E**, **2E**, see arrows) in both plant systems. For RBD, two bands are visible in BY-2 cell packs with a molecular weight of around 25 kDa. In Medicago, at least three bands are detected with sizes between 20 and 30 kDa. In our transient system, the produced RBD protein had a smaller size than its mammalian counterpart.

### RBD is secreted to the culture medium in stably transformed BY-2 and Medicago cell cultures

**Figure 3:**
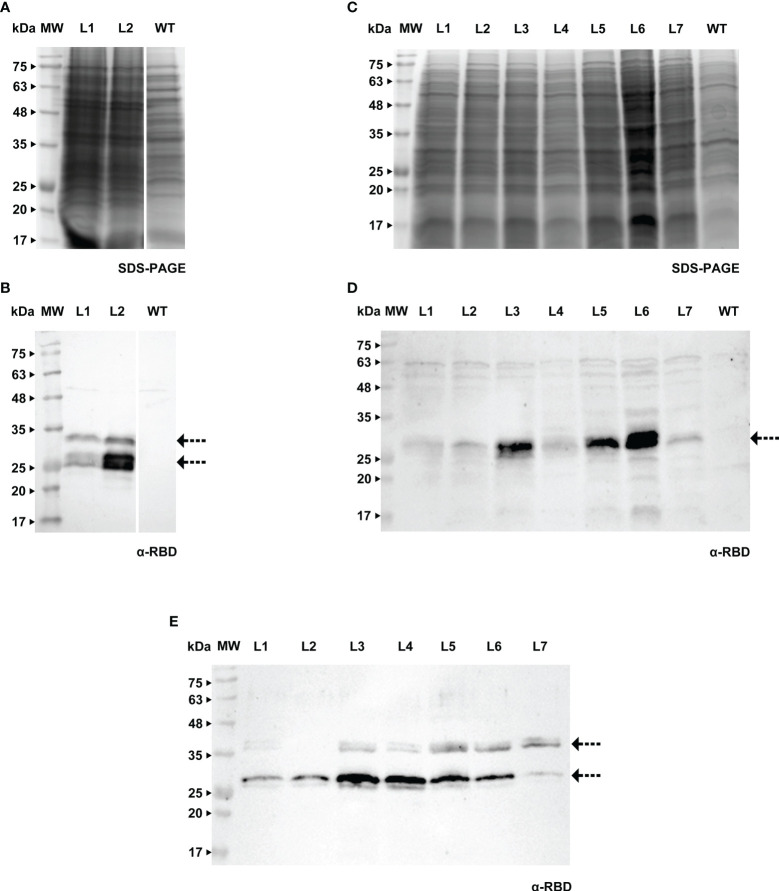
Detection of RBD in *calli* **(A, B)**, cell extracts **(C, D)** and spent medium **(E)** of BY-2. **(A)** Stained gel containing total protein extract of two *calli* of BY-2, WT - wild type. **(B)** Corresponding WB with anti-RBD antibody. **(C)** Stained gel containing cellular extract of seven independent BY-2 cell lines, WT - wild type. **(D)** Corresponding WB with anti-RBD antibody. **(E)** WB analysis of the spent medium of seven independent BY-2 cell lines with anti-RBD antibody.

**Figure 4:**
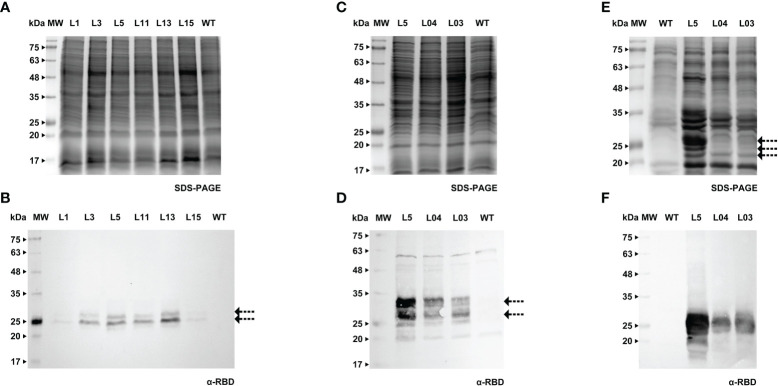
Detection of RBD in *calli* **(A, B)**, cell extracts **(C, D)** and spent medium **(E, F)** of Medicago. **(A)** Stained gel containing total protein extract of six *calli* of Medicago, WT - wild type. **(B)** Corresponding WB with anti-RBD antibody. **(C)** Stained gel containing cellular extract of three independent Medicago cell lines, WT - wild type. **(D)** Corresponding WB with anti-RBD antibody. **(E)** Stained gel containing spent medium of three independent Medicago cell lines, WT - wild type. Samples were concentrated with ice-cold ethanol (L5 - 10x, L04 and L03 - 20x). **(F)** Corresponding WB with anti-RBD antibody.

Cell cultures of tobacco BY-2 and Medicago A17 were stably transformed by Agrobacterium-mediated transfer, using protocols that are well established in our laboratory. Recombinant products were detected in cell extracts and spent medium by western blotting using a specific antibody against RBD (**Figures 3**, **4**).

We were able to detect RBD in *calli* of both species (verified by western blotting, **Figures 3A, B** and **4A, B**), therefore we proceeded to the establishment of liquid cultures. In liquid cultures, RBD was detected in the cell extracts (**Figures 3C, D**, **4C, D**) as well as the spent medium (**Figures 3E**, **4E, F**), revealing that the recombinant protein was secreted as expected. Even though Medicago cultured cells producing RBD were established more recently, SDS-PAGE analysis of the spent medium revealed the presence of the recombinant product, which was not visible in the WT. Subsequent concentration of the spent medium by 10-fold allowed for a clearer identification of a strong band around 25 kDa (**Figure 4E**, L5, see arrows).This was corroborated by western blotting, **Figures 3E** (BY-2) and **4F** (Medicago), showing several putative glycoforms. RBD produced in Medicago showed a lower molecular weight, when compared with BY-2.

The purification strategy for recombinant RBD from BY-2 spent medium was performed by nickel-affinity, relying on the 6xHis tag present at the C-terminal. However, this method proved unsuccessful since RBD was found in the flow-through (**Supplementary Material S3A**). We hypothesize that RBD lost the histidine-tag in BY-2, since we were also not able to detect the recombinant products with an anti-His tag antibody (**Supplementary Material S3B**), although the RBD protein had been already detected in lines 1 to 7 (**Figure 3E**). This is in agreement with what was reported in other plant systems, namely the work by Strasser and colleagues (Shin et al., 2021).

In BY-2, we further investigated whether RBD produced in cultured cells formed a dimer, as reported for *N. benthamiana* and animal cells. We did not detect any dimer when spent medium samples were run under reducing vs. non-reducing conditions (**Supplementary Material S4A, B**). This was further confirmed with a sample of RBD produced in HEK cells (provided by R. Castro). This dimer had a molecular weight between 63 kDa and 75 kDa (**Supplementary Material S4C**).

### Spike protein was successfully produced in cell cultures of BY-2 and Medicago A17

**Figure 5:**
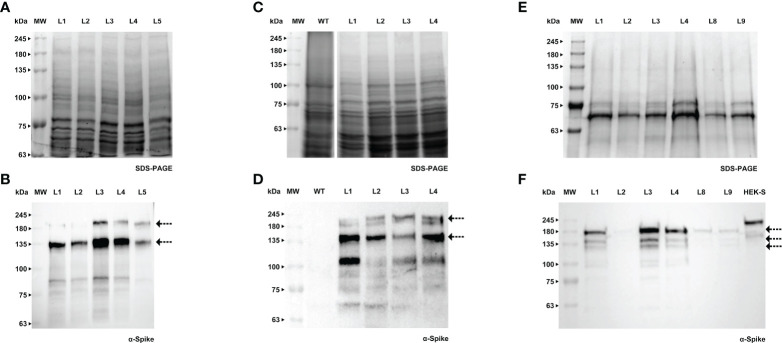
Detection of Spike recombinant protein in *calli* **(A, B)**, cell extracts **(C, D)** and spent medium **(E, F)** of BY-2. **(A)** Stained gel containing total protein extract of five *calli* of BY-2. **(B)** Corresponding WB with anti-Spike antibody. **(C)** Stained gel containing cellular extract of four independent BY-2 cell lines, WT - wild type. **(D)** Corresponding WB with anti-Spike antibody. **(E)** Stained gel containing spent medium of six independent BY-2 cell lines. Samples were concentrated with a centrifugal filter (L1 - 23x, L2 - 14x, L3 - 19x, L4 - 39x, L8 – 17x, L9 – 13x). **(F)** Corresponding WB with anti-Spike antibody.

**Figure 6:**
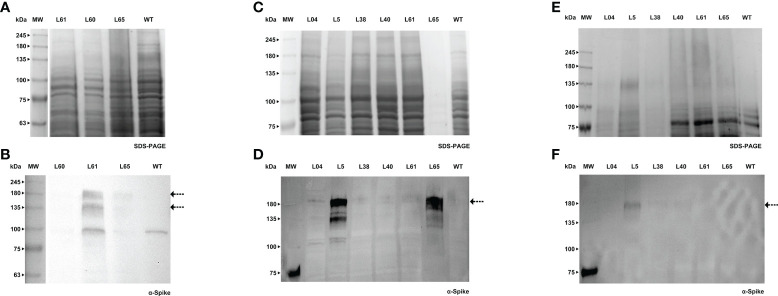
Detection of Spike recombinant protein in *calli* **(A, B)**, cell extracts **(C, D)** and spent medium **(E)** of Medicago. **(A)** Stained gel containing total protein extract of three *calli* of Medicago, WT - wild type. **(B)** Corresponding WB with anti-Spike antibody. **(C)** Stained gel containing cellular extract of six independent Medicago cell lines, WT - wild type. **(D)** Corresponding WB with anti-Spike antibody. **(E)** Stained gel containing spent medium of six independent Medicago cell lines with anti-Spike antibody, WT – wild type. Samples were concentrated with a centrifugal filter (L04 – 18x, L5 – 38x, L38 – 38x, L40 – 32x, L61 – 27x, L65 – 16x). **(F)** Corresponding WB anti-Spike antibody.

Forty days after the transformation event, we were able to detect the S protein in BY-2 *calli* (**Figures 5A, B**). Positive *calli* were selected and liquid cultures were established. In the following week, the recombinant Spike was detected in the cell extract, while the detection of the secreted S protein, which was our main goal, was achieved one week later. For both *calli* and cell extracts, we detected two bands with an anti-Spike antibody, with a molecular weight around 135 and 180 kDa (**Figures 5C, D**, arrows). The recombinant Spike was detected in BY-2 spent medium (**Figures 5E, F**, arrows), with three visible bands on the western blot. We also detected Spike in Medicago A17 *calli* (**Figures 6A, B**) and proceeded to generate liquid cultures. A preliminary assessment of cellular extracts (**Figures 6C, D**) and spent medium (**Figures 6E, F**) in Medicago indicated that the Spike is present in both. Since the Medicago lines were recently established, it is expected that secretion will improve along time, as we have witnessed with several other recombinant proteins in previous studies. We were able to see a signal in the western blot with an anti-Spike, at 180 kDa (**Figure 6F**, arrow).

**Figure 7:**
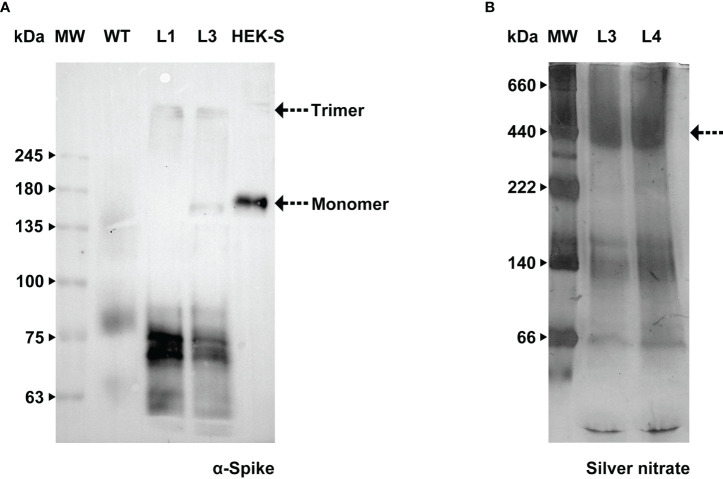
Assessment of Spike native conformation in BY-2 cell lines. **(A)** WB analysis with anti-Spike antibody of total protein extract, carried out under non-reducing conditions, from two independent BY-2 cell lines (L1 e L3), WT – wild type, HEK-S -Spike produced in HEK cell lines (Castro et al., 2021). **(B)** Stained gel containing spent medium of two independent BY-2 cell lines (L3 e L4) carried out under native conditions.

We carried out further investigation on BY-2 cultures in order to detect the Spike trimer and assess whether this production platform was able to assemble and secrete such a complex protein. Total protein extract from BY-2 cell lines producing the recombinant Spike and the purified HEK-produced S protein (Castro et al., 2021) were resolved in a 7% polyacrylamide gel. Samples were added to the sample buffer without the presence of reducing agents and were not boiled, to preserve disulfide bonds. We firstly detected the trimer in the cell extract, similarly to what has been reported in *N. benthamiana* leaves (Jung et al., 2022). WB analysis (**Figure 7A**) detected two bands in the HEK-S sample, corresponding to the glycosylated monomer, around 180 kDa, and a higher molecular weight band that may correspond to the trimer. Castro and coworkers showed the presence of the trimer in the purified sample by HPLC-SEC.

We undertook further efforts to detect a tertiary structure in native conditions. Concentrated spent medium from BY-2 cell lines 3 and 4 (19- and 39-fold respectively) were resolved in 5-15% Bis-Tris polyacrylamide gel. No reducing agent was added and the ionic detergent SDS was exchanged for a non-ionic surfactant (n-dodecyl β-D-maltoside, DDM). Since Native-PAGE gel stained with Coomassie G-250 did not detect high molecular weight proteins, we proceeded with silver nitrate staining. Upon increasing sensitivity in total protein detection, we detected a band around 500 kDa in the culture medium (**Figure 7B**). This is in agreement with Fernandes and co-workers who carried out HPLC-SEC and obtained chromatograms showing only one main form of S protein with approximately 400 kDa (Fernandes et al., 2022). The aggregates reported by Fernandes and colleagues could justify our finding in the western blot performed after running the sample on a 5% Native-PAGE. A signal with an anti-Spike antibody was detected in the region of the stacking gel, showing that the protein did not migrate into the resolving gel (**Supplementary Material S5**).

### Purification of Spike protein from BY-2 cell extracts and glycoanalysis

**Figure 8:**
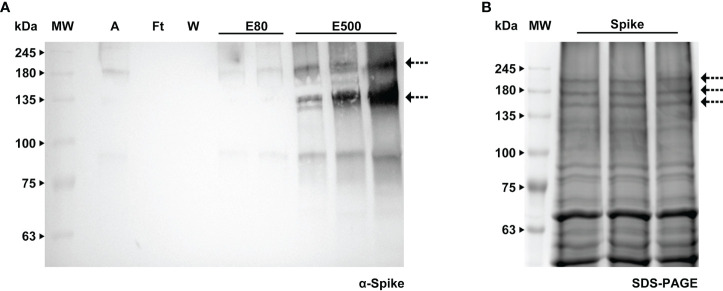
Purification of Spike protein by immobilized metal affinity using a HisTrap™ High Performance column. **(A)** WB analysis of the imidazole eluted fractions of Spike protein with anti-Spike antibody, A - sample, Ft - flow through, W - wash, E80 - and E500 - eluted fractions. **(B)** Stained gel containing the concentrated eluted fractions after desalting; the bands corresponding to the Spike protein (indicated by an arrow) were further analyzed by MS.

Contrarily to RBD, the Spike protein maintained the His-tag, thus we carried out purification and were able to obtain a partially cleaned product which was sent for MS analysis to confirm identity. The procedure consisted of passing the total protein extract on a His-trap column and the two fractions that contained the Spike (**Figure 8A**) were desalted using a PD-10. These fractions were then run through an Amicon-15, leading to a 17-fold concentrated sample (arrow in **Figure 8B**).

The bands were extracted from the gel and sent to MS analysis for determination of identity and glycoprofiling. *A Nicotiana tabacum* database was used to compare the results obtained in the mass spectra of our sample. The identity of the Spike protein was confirmed with the finding of 120 peptides above 95% confidence. For glycoanalysis, a protein sequence coverage of about 50% was obtained. For peptide assignment, an m/z tolerance of 5 ppm was considered and the glycans were identified according to the databased described in Materials and methods. 13 N-glycosylation sites (out of 22 identified in Watanabe et al., 2020) were analyzed and compared with the human-produced Spike described in Castro et al. (2021). Glycoforms of each identified site were compared based on the presence of high mannose, fucosylated, xylosylated, or xylosylated and fucosylated motifs. This analysis is shown schematically in **Table 2**.

**Table 2:** Comparison of glycan composition between the recombinant Spike produced in BY-2 cells and the Spike produced in human HEK293-E6 cells (Castro et al., 2021).

Glycan	N4		N48		N109		N136		N152		N221		N269		N318		N785		N1058		N1082		N1157		N1178	
			N61												N321											
	HEK	BY-2	HEK	BY-2	HEK	BY-2	HEK	BY-2	HEK	BY-2	HEK	BY-2	HEK	BY-2	HEK	BY-2	HEK	BY-2	HEK	BY-2	HEK	BY-2	HEK	BY-2	HEK	BY-2
1																										
2																										
3																										
4																										
5																										
6																										
7																										
8																										
9																										
10																										
11																										
12																										
13																										
14																										
15																										
16																										
17																										
18																										
19																										
20																										
21																										
22																										
23																										
24																										
25																										
26																										
27																										
28																										
29																										
30																										
31																										
32																										
33																										
34																										
35																										
36																										
37																										
38																										
39																										
40																										
41																										
42																										
43																										
44																										
45																										
46																										
47																										
48																										
49																										
50																										
51																										
52																										
53																										
54																										
55																										
56																										
57																										
58																										
59																										
60																										
61																										
62																										
63																										
64																										
65																										
66																										
67																										
68																										
69																										
70																										
71																										
72																										
73																										
74																										
75																										
76																										
77																										
78																										
79																										
80																										
81																										
82																										
83																										
84																										
85																										
86																										
87																										
88																										
89																										
90																										
91																										
92																										
93																										
94																										
95																										
96																										
97																										
98																										
99																										
100																										
101																										
102																										
103																										
104																										
105																										
106																										
107																										
108																										
109																										
110																										
111																										
112																										
113																										
114																										
115																										

MS data were screened considering the 69 N-glycan structures previously identified (**Supplementary Material S2**). Colors represent the following N-glycans structural features: High mannose (green), fucosylated (red), sialylated (purple), sialylated/fucosylated (dark gray), sialyl-LacdiNac (yellow), and others (light gray).

## Discussion

Recombinant viral glycoproteins are generally poorly processed and often accumulate at low levels in plants (Shin et al., 2021). N-Glycosylation and associated quality control processes have a major role in the effective production of these complex recombinant proteins (Shin et al., 2021). To date, the production of SARS-CoV-2-related recombinant proteins in plant systems has been only achieved by transient expression in *N. benthamiana* plants. In this work, we engineered cell suspension cultures of tobacco and Medicago to produce the soluble Spike protein and the RBD of SARS-CoV-2. We showed that cell cultures are a valid platform for the expression of large, complex proteins, which can be secreted into the culture medium from where they can be purified without the need for cell disruption procedures. Furthermore, the production is continuous and fully contained, facilitating compliance with good manufacturing practices.

We firstly produced both Spike and RBD in a transient system using the cell pack method described in Rademacher et al. (2019). For Medicago cell cultures, this procedure needed optimization as this species produces cell aggregates and is relatively different in liquid culture when compared with tobacco BY-2 cells. This method is a rapid and versatile expression system that involves the infusion of Agrobacterium suspension into three-dimensional, porous plant cell aggregates deprived of cultivation medium (Rademacher et al., 2019). The recombinant proteins were produced by both species in this screening, indicating that cultured cells were able to express the desired proteins. Nonetheless, the Spike protein presented more degradation in BY-2 than in Medicago, in accordance with our previous data showing that Medicago has a lower proteolytic activity than BY-2 (Santos et al., 2018).

Following successful transient expression, we then transformed both BY-2 and Medicago A17 cultures by Agrobacterium-mediated gene transfer. *Calli* which were actively growing under selection were tested for the presence of either RBD or Spike, revealing multiple bands by western blotting, which is in agreement with the early stages of transformed cells when many glycoforms are detected corresponding to various intracellular and extracellular accumulation sites.

Upon establishment of liquid cultures from selected *calli*, samples of the spent medium were collected at 2-week intervals and evaluated by western blotting (results not shown). In the first weeks, we observed that the amount of secreted recombinant RBD was variable, but it became stable and increased rapidly. For the Spike protein, which has a molecular weight of around 540 kDa, the production was expected to take longer to stabilize; nevertheless, we were able to detect the protein 40 days after co-culture. It is noteworthy that our transgenic lines followed a similar growth rate as the wild type, which has a doubling time of around 24h (Santos et al., 2016). In order to obtain higher levels of recombinant product, an industrial-scale process should be implemented. This can be challenging since several factors can influence the cell culture behavior, when transferring the production from the laboratory to the industrial scale. Although our study was only performed at the bench scale (2 L), we envision that it would be possible to increase to a pilot-scale (50 L) in 2 weeks, starting from one only Erlenmeyer with 50 mL of culture. For a larger scale, industrial bioreactors would be used instead of shake-flasks. In any case, we can estimate that we could obtain 2000 L from a 50 mL culture within 4 weeks at the bench level, as we use a 3% inoculum.

As BY-2 cultures were engineered several months before Medicago, we started by carrying out analyses of the recombinant products in BY-2 cell lines. As reported by other authors (Shin et al., 2021 and references therein), we confirmed that RBD lost the C-terminal part including the histidine-tag (**Supplementary Material S3**). The protein was detected using an anti-RBD antibody but not detected when using an anti-His tag. Accordingly, when we attempted purification with a His-trap column, all RBD was found in the flow-through, indicating that it did not bind to the nickel resin. In fact, our plant produced RBD showed two bands (with a stronger intensity) that were smaller than the human-produced counterpart and one higher band that is similar to the human produced RBD. We further investigated whether we could see a homodimer as reported by other authors. For this, we performed a comparison of the RBD from BY-2 using reducing vs. non-reducing conditions. In our hands, RBD did not form a dimer. Authors argue that this dimer can be due to the presence of several cysteine residues in RBD that can cause the formation of disulfide bonds and protein aggregation (Shin et al., 2021). It should be noted that RBD expression induced necrosis in infiltrated leaves (Maharjan et al., 2021) and is reported to be likely toxic to plant cells. We did not observe any impairment of the growth of transgenic cell lines transformed with the RBD coding sequence. Although the quantification of the products was not performed, Medicago cells achieved higher yields of recombinant RBD than BY-2 cells in a much shorter timeframe.

The modified version of the SARS-CoV-2 Spike full-length protein is composed of 3 monomers of around 140 kDa each, 180 kDa with glycans, indicating that the trimer is around 540 kDa. In plant cells, the production of recombinant proteins with such a high molecular weight is not common and can constitute an issue for protein secretion to the culture medium. For example, the human butyrylcholinesterase (BChE) is a complex and heavily glycosylated protein comprised of four identical 85 kDa monomers. Expression of BChE in rice cell suspension cultures revealed that protein secretion to the culture medium, although detected during low-scale screening production, was heavily decreased during scale-up bioreactor operations (Corbin et al., 2016). Thus, it is a potential challenge to achieve an efficient secretion of such high molecular weight proteins.

Difficulties in obtaining stable, soluble S protein trimers have been reported in mammalian cells, because of their tendency to disassemble upon purification and storage leading to a combination of unprocessed precursor and cleaved forms, likely including free S1, S2 and S1/S2 complexes (Stuible et al., 2021). In our study, we used a modified sequence of the Spike gene that generates a stabilized soluble unprocessed protein, including a mutated furin site and a trimerization domain fusion. This sequence has been used for production of Spike in human and insect cells (Amanat et al., 2020; Castro et al., 2021; Fernandes et al., 2022). In our study, the only modification performed was a codon-optimization for plant expression. Although plants do not show furin cleavage activity, we observed some proteolysis of the full-length Spike. Ongoing work in our laboratory using the native wild-type sequence of the Spike protein will enable a better understanding of the intracellular processing as well as stability of the Spike protein.

Fernandes and co-workers assessed S protein aggregation using HPLC-SEC, which the authors claim to be one of the more robust and reproducible methods for tracing protein aggregates. Their chromatograms showed one main form of S protein with approximately 400 kDa, consistent with its tertiary structure. If we were able to purify Spike from the culture medium and perform HPLC-SEC, we would likely be able to confirm the presence of the trimer. In fact, conventional methods such as gel filtration or Native-PAGE present technical difficulties, which is probably why most reports on trimeric S protein rely on HPLC-SEC.

Recombinant Spike protein has a wide range of potential applications, including the development of novel therapeutics, clinical diagnostic tools or subunit vaccines. Glycosylation of viral proteins plays an important role in their folding, virus entry, and the host immune response to infection (reviewed in Ruocco and Strasser, 2022). Contrary to mammalian and plant cells, bacterial expression systems lack post-translation modifications and have limited disulfide-bond formation (Chuck et al., 2009; Fitzgerald et al., 2021) which can lead to incorrect folding and lower protein stability or activity (Liu et al., 2022). To our knowledge, there is only one report of the full-length Spike protein produced in bacteria (Wang et al., 2020). Here, the authors report the display of the recombinant Spike at the surface of *Lactobacillus plantarum*, suggesting this could be a candidate to an oral vaccine. All the other reports available in the literature refer to partial sequences of S protein, such as fragments (McGuire et al., 2022) and RBD (Fitzgerald et al., 2021; Maffei et al., 2021; Prahlad et al., 2021; Liu et al., 2022; Matsunaga and Tsumoto, 2022). Prahlad et al. (2021) reported that bacterial RBD was able to interact with ACE2 even though with lower affinity, and Maffei et al. (2021) observed lower thermal stability, when compared to RBD produced in eukaryotic systems.

Glycosylation is an important parameter in antigen design for subunit vaccine development or for components in serological diagnostic tests (Ruocco and Strasser, 2022). Our preliminary analysis of the glycan profile indicates that Spike contains the same N-glycosylation sites as the one produced in human cells, however with several differences including plant-specific glycans such as β-1,2-xylose and α-1,3-fucose. The effect of these specificities on the binding ability (for example to neutralizing antibodies) of the recombinant Spike will be the subject of further investigation, once purification is achieved on a larger scale.

## Conclusion

This study demonstrates the potential of plant cell suspension cultures for the expression of SARS-CoV-2 S protein and its RBD to be used as reagents in diagnostics and therapeutics. RBD was produced in considerably larger amounts than Spike but it lost the C-terminal histidine-tag, hampering its purification procedure. The loss of the C-terminal part of the RBD has been reported by other authors who carried out transient expression in *N. benthamiana* as discussed above. Inversely, the Spike protein kept the His-tag and was further purified and subjected to glycosylation analysis and comparison with its human cell produced counterpart. Regarding folding and integrity, although some cleavage products were obtained, we were able to detect a band in the range of 500 kDa on a BN gel, indicating that trimeric Spike was produced and secreted to the culture medium.

We will further improve our system with the addition of histone deacetylase inhibitors as we have previously done with a human prostaglandin D synthase in Medicago cell lines (Santos et al., 2017) and BY-2 (Rebelo et al., 2020) and also in line with protocols used in animal cells for recombinant Spike production (Castro et al., 2021). On the other hand, ongoing work in our group on the use of the native Spike sequence, containing the furin cleavage site, will provide further information on how plant cells process large proteins. To date, no furin cleavage activity has been observed in plants, thus it is expected that the trimer will be produced without the need to use a modified sequence such as the one generated by F. Krammer’s group used in this study. The impact of the plant protease repertoire on viral glycoprotein production in plants is unknown (Margolin et al., 2022). However, there is an abundance of proteases along the plant secretory pathway, which could have a negative effect on glycoprotein accumulation. For this reason, since we have previously demonstrated that the proteolytic activity of *Medicago truncatula* culture medium is significantly lower compared with BY-2 (Santos et al., 2018), we believe that our continued studies on Medicago cell cultures will lead to better yields. This is also in accordance with our previous work on the production of recombinant Phytase (molecular weight of 70 kDa), for which Medicago performed better than tobacco (Abranches et al., 2005; Pires et al., 2008).

Collectively, our data demonstrate that plant cell cultures can be a suitable platform for the production of complex viral proteins with various potential applications in the combat against emerging diseases.

## Data availability statement

The original contributions presented in the study are included in the article/**Supplementary Material**. Further inquiries can be directed to the corresponding author.

## Author contributions

RA conceptualized the study with input from AF and BAR. BAR, AF and ACF conducted experiments. RA drafted the manuscript. All authors contributed to data analysis and approved the manuscript.

## Funding

This work was supported by Fundação para a Ciência e Tecnologia (FCT, Portugal) through Research Units “Molecular, Structural and Cellular Microbiology” (MOSTMICRO, UIDB/04612/2020) and “Bioresources 4 Sustainability” (GREEN-IT, UIDB/04551/2020).

## Acknowledgments

The authors wish to thank Rute Castro for helpful discussions throughout the course of this work and for providing samples of recombinant RBD and Spike proteins to be used as controls, Cristina Timóteo for helping with the purification of recombinant proteins, João Vicente and Américo Duarte for helpful technical advice, and Ricardo Gomes and Bruno Alexandre for help with the MS analysis. MS data were obtained by the UniMS—Mass Spectrometry Unit, ITQB/IBET, Oeiras, Portugal. Last and foremost, we thank all the colleagues from the ITQB NOVA Covid Taskforce for fruitful discussions and sharing of reagents and materials.

## Conflict of interest

The authors declare that the research was conducted in the absence of any commercial or financial relationships that could be construed as a potential conflict of interest.

## Publisher’s note

All claims expressed in this article are solely those of the authors and do not necessarily represent those of their affiliated organizations, or those of the publisher, the editors and the reviewers. Any product that may be evaluated in this article, or claim that may be made by its manufacturer, is not guaranteed or endorsed by the publisher.
