# Supplementary material for: Production of the SARS-CoV-2 Spike protein and its Receptor Binding Domain in plant cell suspension cultures
Source: Front Plant Sci. 2022 Oct 21;13:995429. doi: 10.3389/fpls.2022.995429 (PMC9634662; doi:10.3389/fpls.2022.995429)

Supplementary Material

Supplementary Material S1 | Nucleotide sequences of the receptor binding domain (RBD, aminoacids 319-541) and the full-length stabilized version of the Spike protein (GeneBank: MN908947.3). Restriction enzymes used for the plasmid construction are marked in bold.

<u>Target sequence: RBD</u>

CCATGGGTAGAGTGCAACCTACAGAATCAATTGTGAGGTTTCCAAATATAACTAACTTG
TGCCCATTCGGAGAAGTGTTCAACGCTACAAGGTTCGCATCAGTGTACGCATGGAATAG
AAAAAGAATATCAAATTGCGTTGCTGATTACTCTGTGCTTTATAATTCAGCATCTTTCTC
AACTTTTAAATGTTATGGAGTTTCACCAACAAAATTGAATGATCTTTGTTTTACTAATGT
TTACGCAGACTCTTTCGTGATTAGGGGAGACGAGGTGAGGCAGATAGCTCCCGGACAGA
CTGGAAAGATTGCAGATTACAATTATAAGCTTCCAGATGACTTCACTGGATGCGTGATT
GCTTGGAATTCTAATAATTTGGACTCTAAAGTTGGTGGAAACTATAATTACCTTTATAGA
TTGTTTAGAAAGTCTAATTTGAAACCATTCGAAAGGGATATATCTACAGAAATTTACCA
AGCTGGATCTACACCTTGCAACGGAGTGGAGGGTTTCAACTGCTATTTCCCTCTTCAATC
TTATGGATTTCAACCTACTAATGGAGTTGGATACCAACCATATAGAGTGGTGGTGTTGTC
TTTCGAGTTGTTGCACGCTCCAGCTACAGTGTGTGGACCTAAAAAGTCTACTAACCTTGT
TAAGAATAAATGCGTTAACTTT**GTCGAC**

<u>Target sequence: Spike</u>

CCATGGGTCAGTGTGTGAATCTTACAACTAGGACACAGCTTCCACCAGCTTACACAAAC
TCATTCACTAGAGGTGTGTACTATCCAGATAAGGTGTTTAGATCTTCTGTTTTGCATTCA
ACTCAAGACTTGTTTCTTCCATTTTTCTCTAATGTTACTTGGTTTCATGCTATTCATGTGTC
TGGTACAAATGGTACTAAAAGATTCGACAATCCAGTGTTGCCTTTCAACGACGGTGTGT
ATTTCGCTTCTACAGAAAAATCAAACATTATAAGGGGTTGGATTTTTGGTACTACTCTTG
ATTCTAAAACACAGTCATTGTTGATTGTTAATAACGCTACAAACGTGGTTATTAAGGTTT
GTGAGTTTCAGTTTTGTAATGATCCTTTTTTGGGAGTTTACTACCATAAAAATAATAAGT
CTTGGATGGAATCTGAATTTAGAGTGTACTCTTCTGCAAACAATTGTACTTTTGAATATG
TTTCTCAGCCATTCCTTATGGACCTTGAGGGTAAGCAAGGAAACTTTAAGAACTTGAGG
GAATTTGTTTTTAAAAATATTGATGGATATTTCAAAATATACTCAAAGCATACACCAATA
AACCTTGTGAGGGACCTTCCACAAGGATTTTCTGCACTTGAGCCTCTTGTGGATTTGCCT
ATTGGAATTAATATTACAAGGTTCCAGACTTTGTTGGCTCTTCATAGATCTTACCTTACTC
CCGGAGACTCTTCATCTGGTTGGACAGCTGGAGCAGCTGCTTACTACGTTGGATACTTGC
AGCCAAGAACTTTCCTTTTGAAGTACAATGAGAATGGAACTATTACTGATGCTGTGGAC
TGCGCTCTTGACCCTCTTTCTGAGACTAAGTGCACTCTTAAATCTTTCACTGTTGAGAAA
GGTATATATCAGACATCTAACTTCAGAGTGCAACCTACAGAATCAATTGTGAGGTTTCC
AAATATAACTAACTTGTGCCCATTCGGAGAAGTGTTCAACGCTACAAGGTTCGCATCAG
TGTACGCATGGAATAGAAAAAGAATATCAAATTGCGTTGCTGATTACTCTGTGCTTTATA

ATTCAGCATCTTTCTCAACTTTTAAATGTTATGGAGTTTCACCAACAAAATTGAATGATC
TTTGTTTTACTAATGTTTACGCAGACTCTTTCGTGATTAGGGGAGACGAGGTGAGGCAGA
TAGCTCCCGGACAGACTGGAAAGATTGCAGATTACAATTATAAGCTTCCAGATGACTTC
ACTGGATGCGTGATTGCTTGGAATTCTAATAATTTGGACTCTAAAGTTGGTGGAAACTAT
AATTACCTTTATAGATTGTTTAGAAAGTCTAATTTGAAACCATTCGAAAGGGATATATCT
ACAGAAATTTACCAAGCTGGATCTACACCTTGCAACGGAGTGGAGGGTTTCAACTGCTA
TTTCCCTCTTCAATCTTATGGATTTCAACCTACTAATGGAGTTGGATACCAACCATATAG
AGTGGTGGTGTTGTCTTTCGAGTTGTTGCACGCTCCAGCTACAGTGTGTGGACCTAAAAA
GTCTACTAACCTTGTTAAGAATAAATGCGTTAACTTTAATTTTAATGGTCTTACTGGAAC
TGGTGTTTTGACTGAATCAAACAAAAAGTTCCTTCCTTTTCAGCAGTTCGGAAGGGACAT
AGCAGACACTACTGACGCAGTGAGAGACCCACAAACTCTTGAGATTTTGGATATAACTC
CTTGTTCATTCGGTGGAGTTTCTGTTATTACACCCGGAACAAACACTTCTAACCAAGTTG
CTGTGCTTTACCAAGATGTGAACTGTACAGAGGTGCCAGTGGCTATTCACGCTGACCAG
TTGACACCAACTTGGAGAGTTTATTCTACTGGATCAAACGTGTTCCAGACTAGGGCTGG
ATGCCTTATTGGTGCAGAACATGTGAATAATTCTTATGAATGTGATATTCCAATTGGTGC
TGGAATATGCGCTTCATACCAGACACAGACAAACTCTCCAGCATCAGTGGCATCACAGT
CTATTATTGCTTACACAATGTCTTTGGGAGCAGAGAACTCTGTGGCTTACTCTAACAATT
CTATTGCTATACCAACTAACTTCACTATTTCTGTGACAACTGAAATATTGCCAGTGTCAA
TGACTAAGACTTCAGTTGATTGCACTATGTACATTTGTGGAGATTCAACAGAGTGTTCTA
ACTTGTTGCTTCAATACGGATCATTTTGTACTCAACTTAACAGAGCTCTTACTGGTATTG
CTGTGGAGCAAGATAAGAACACTCAAGAAGTTTTCGCACAAGTTAAGCAAATTTATAAG
ACACCTCCAATTAAGGATTTTGGTGGATTCAACTTCTCTCAGATACTTCCAGACCCTTCA
AAACCTTCTAAAAGATCATTCATTGAAGATTTGTTGTTCAATAAAGTGACACTTGCAGAC
GCTGGATTCATAAAGCAGTACGGTGACTGTTTGGGTGACATAGCTGCAAGGGACTTGAT
ATGCGCACAGAAATTCAACGGTTTGACAGTGTTGCCTCCACTTTTGACTGACGAGATGAT
TGCACAATATACTTCTGCACTTTTGGCTGGTACAATTACATCTGGATGGACATTCGGTGC
TGGAGCAGCTCTTCAAATTCCTTTCGCAATGCAAATGGCTTATAGGTTTAATGGTATTGG
TGTGACTCAAAATGTGCTTTATGAGAATCAAAAACTTATTGCAAACCAATTTAATTCTGC
AATTGGTAAAATACAAGATTCTTTGTCATCAACAGCATCTGCTCTTGGAAAGTTGCAAG
ATGTGGTGAACCAGAACGCACAAGCTTTGAACACTCTTGTGAAGCAACTTTCATCAAAC
TTTGGAGCAATATCATCTGTTCTTAACGATATTCTTTCTAGGCTTGACCCTCCAGAAGCA
GAAGTTCAGATTGATAGATTGATTACTGGAAGATTGCAATCACTTCAAACTTATGTTACT
CAGCAGCTTATTAGGGCTGCAGAGATAAGGGCATCTGCTAACCTTGCAGCTACTAAGAT
GTCTGAGTGCGTGCTTGGTCAATCTAAAAGGGTGGATTTCTGCGGTAAGGGATATCACC
TTATGTCTTTCCCTCAGTCAGCTCCTCACGGTGTGGTGTTCTTGCACGTGACTTACGTGCC
AGCTCAAGAAAAGAACTTCACTACTGCACCAGCTATTTGCCATGACGGTAAGGCACACT
TCCCAAGAGAGGGTGTGTTCGTGTCAAACGGAACACACTGGTTCGTGACTCAAAGAAAT
TTCTATGAGCCACAAATTATTACTACTGACAATACTTTTGTTTCTGGAAATTGCGACGTT
GTGATTGGTATTGTTAACAACACTGTTTACGACCCATTGCAGCCAGAATTGGATTCTTTT
AAGGAAGAATTGGATAAATACTTCAAGAATCACACTTCTCCAGATGTTGATTTGGGTGA
TATTTCTGGAATAAATGCATCAGTGGTTAACATTCAAAAGGAGATAGATAGGCTTAATG
AAGTGGCAAAGAATCTTAATGAATCTCTTATTGATCTTCAAGAGCTTGGTAAATACGAA
CAATATATAAAGTGGCCATCTGGTAGGTTGGTGCCTAGGGGATCTCCCGGATCTGGATA
CATTCCAGAAGCACCTAGGGACGGACAAGCTTACGTTAGGAAGGACGGAGAGTGGGTT
CTTCTTTCAACATTCTTGGGA**GTCGAC**

Supplementary Material S2 | List of N-glycan composition screened in Spike protein MS analysis. The database is composed of 69 types of glycans.

Note: For glycosylation comparison between human and plant recombinant Spike, the numeric order of the 109 glycans identified by Castro et al. (2021) was maintained, and the 6 glycans identified in plants were added from position No. 110 to No. 115. For the screening, all glycans containing sialylation were removed.

Nº	COMPOSITION	Nº	COMPOSITION	Nº	COMPOSITION
**1**	HexNAc(2)Hex(9)	**31**	HexNAc(4)Hex(4)Fuc(2)	**80**	HexNAc(6)Hex(3)Fuc(2)
**2**	HexNAc(2)Hex(8)	**33**	HexNAc(4)Hex(5)	**81**	HexNAc(6)Hex(3)Fuc(3)
**3**	HexNAc(2)Hex(7)	**34**	HexNAc(4)Hex(5)Fuc(1)	**82**	HexNAc(6)Hex(4)
**4**	HexNAc(2)Hex(6)	**37**	HexNAc(4)Hex(5)Fuc(2)	**83**	HexNAc(6)Hex(4)Fuc(1)
**5**	HexNAc(2)Hex(5)	**39**	HexNAc(4)Hex(5)Fuc(3)	**84**	HexNAc(6)Hex(4)Fuc(2)
**6**	HexNAc(2)Hex(4)	**43**	HexNAc(4)Hex(6)	**86**	HexNAc(6)Hex(5)
**7**	HexNAc(2)Hex(4)Fuc(1)	**44**	HexNAc(4)Hex(6)Fuc(1)	**87**	HexNAc(6)Hex(5)Fuc(1)
**8**	HexNAc(3)Hex(5)	**46**	HexNAc(4)Hex(6)Fuc(2)	**91**	HexNAc(6)Hex(5)Fuc(2)
**9**	HexNAc(3)Hex(5)Fuc(1)	**47**	HexNAc(4)Hex(6)Fuc(3)	**92**	HexNAc(6)Hex(6)
**11**	HexNAc(3)Hex(6)	**49**	HexNAc(5)Hex(3)	**93**	HexNAc(6)Hex(6)Fuc(1)
**12**	HexNAc(3)Hex(6)Fuc(1)	**50**	HexNAc(5)Hex(3)Fuc(1)	**96**	HexNAc(6)Hex(6)Fuc(2)
**15**	HexNAc(2)Hex(3)	**52**	HexNAc(5)Hex(4)	**97**	HexNAc(6)Hex(6)Fuc(3)
**16**	HexNAc(2)Hex(3)Fuc(1)	**53**	HexNAc(5)Hex(4)Fuc(1)	**98**	HexNAc(6)Hex(7)
**17**	HexNAc(3)Hex(3)	**56**	HexNAc(5)Hex(4)Fuc(2)	**99**	HexNAc(6)Hex(7)Fuc(1)
**18**	HexNAc(3)Hex(3)Fuc(1)	**60**	HexNAc(5)Hex(5)	**107**	HexNAc(7)Hex(3)Fuc(1)
**19**	HexNAc(3)Hex(4)	**61**	HexNAc(5)Hex(5)Fuc(1)	**108**	HexNAc(7)Hex(4)
**20**	HexNAc(3)Hex(4)Fuc(1)	**64**	HexNAc(5)Hex(5)Fuc(2)	**109**	HexNAc(7)Hex(4)Fuc(1)
**23**	HexNAc(4)Hex(3)	**65**	HexNAc(5)Hex(5)Fuc(3)	**110**	HexNAc(2)Hex(3)Xyl(1)
**24**	HexNAc(4)Hex(3)Fuc(1)	**67**	HexNAc(5)Hex(6)	**111**	HexNAc(2)Hex(3)Fuc(1)Xyl(1)
**25**	HexNAc(4)Hex(3)Fuc(2)	**68**	HexNAc(5)Hex(6)Fuc(1)	**112**	HexNAc(3)Hex(3)Xyl(1)
**26**	HexNAc(4)Hex(3)Fuc(3)	**72**	HexNAc(5)Hex(6)Fuc(2)	**113**	HexNAc(3)Hex(3)Fuc(1)Xyl(1)
**28**	HexNAc(4)Hex(4)	**77**	HexNAc(6)Hex(3)	**114**	HexNAc(4)Hex(3)Fuc(1)Xyl(1)
**29**	HexNAc(4)Hex(4)Fuc(1)	**78**	HexNAc(6)Hex(3)Fuc(1)	**115**	HexNAc(4)Hex(3)Xyl(1)

Supplementary Material S3 | Purification of RBD protein by immobilized metal affinity using a HisTrap™ High Performance column. **(A)** WB analysis of the eluted fractions of RBD protein with anti-RBD antibody, A - sample, Ft - flow through, W - wash, E500 - eluted fraction. **(B)** WB analysis of spent media from seven independent BY-2 lines (L1 to L7, samples corresponding to Figure 3E) with anti-His tag antibody.

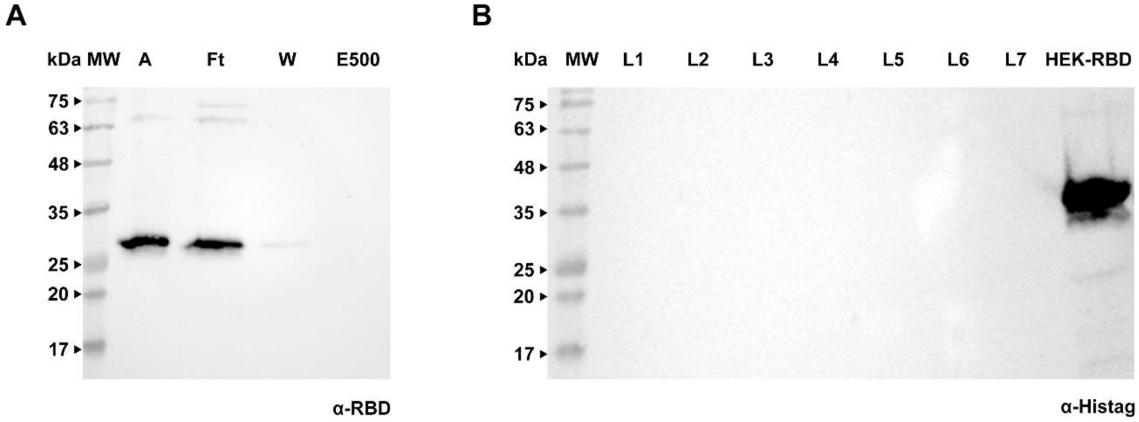

Supplementary Material S4 | Comparison of RBD protein produced in BY-2 and HEK cells under non-reducing conditions. **(A)** Stained gel containing the spent medium of BY-2 cell line 5, L5- reducing conditions, L5 nr- non-reducing conditions. **(B)** Corresponding WB with anti-RBD antibody. **(C)** Stained gel containing the purified RBD from HEK cells, HEK-RBD- reducing conditions, HEK-RBD nr- non-reducing conditions.

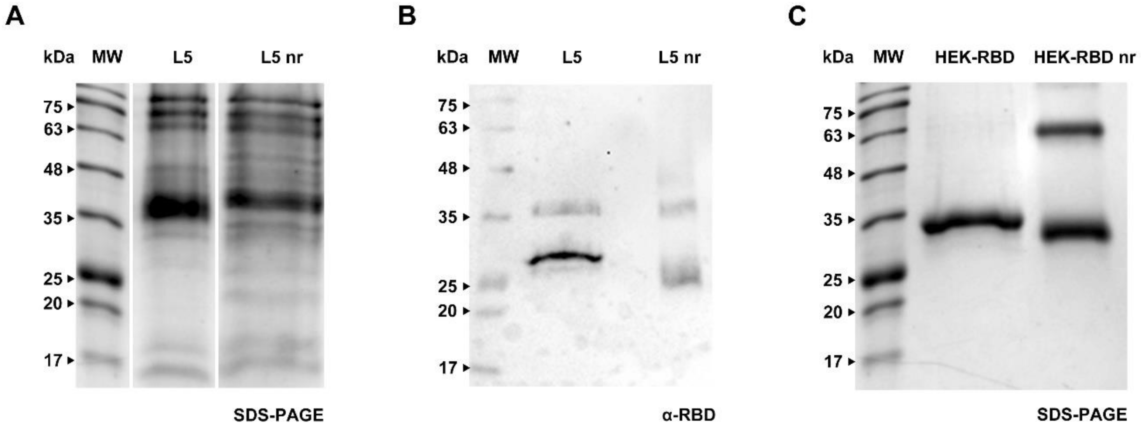

Supplementary Material S5 | Detection of Spike native conformation in BY-2 spent medium. Samples for two independent lines (L3 and L4, same samples as in Figure 7B) were loaded into a 5 % polyacrylamide native gel and subjected to WB detection with anti-Spike antibody.

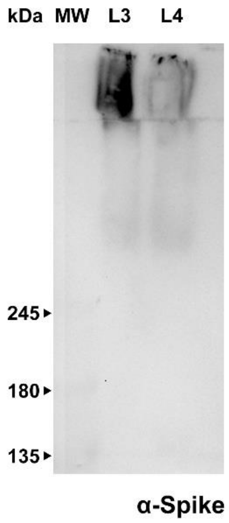

Supplement: Supplementary file 1 [file DataSheet_1.pdf]
